# Supplementary material for: The interactome of intact mitochondria by cross-linking mass spectrometry provides evidence for coexisting respiratory supercomplexes
Source: Mol Cell Proteomics. 2017 Dec 8;17(2):216–32. doi: 10.1074/mcp.RA117.000470 (PMC5795388; doi:10.1074/mcp.RA117.000470)
Supplement: Supplemental Data [file supp_RA117.000470_133922_0_supp_23978_fzffwf.zip › spectra_annotation/mito_DR_spectra_annotation/66-1-5-1-20-1.pdf]

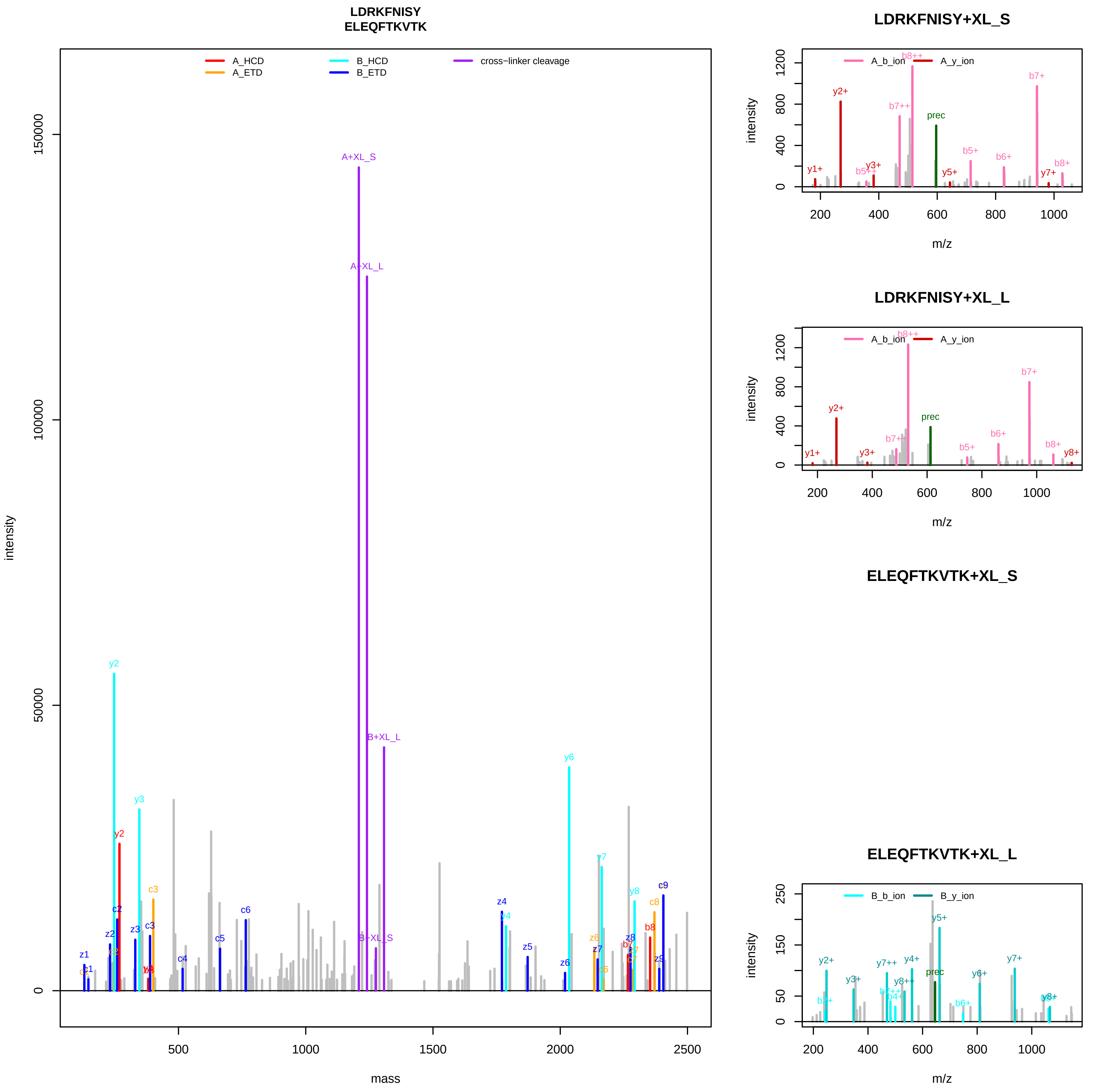

LDRKFNISY
ELEQFTKVTK
A_HCD
A_ETD
B_HCD
B_ETD
cross−linker cleavage
A+XL_S
A+XL_L
B+XL_L
B+XL_S
y2
y3
y6
y7
y8
c2
z2
z3
c3
z1
c1
c4
c5
c6
z4
y4
z5
z6
z7
z8
z9
c7
c8
c9
b7
b8
intensity
mass
0
50000
100000
150000
500
1000
1500
2000
2500
LDRKFNISY+XL_S
A_b_ion
A_y_ion
b8++
y2+
b7++
prec
b7+
y1+
b5++
y3+
b5+
b6+
y5+
b8+
y7+
intensity
m/z
LDRKFNISY+XL_L
b8++
y2+
prec
b7+
y1+
y3+
b7++
b5+
b6+
b8+
y8+
ELEQFTKVTK+XL_S
ELEQFTKVTK+XL_L
B_b_ion
B_y_ion
y5+
y2+
y3+
y7++
y4+
y8++
prec
y6+
y7+
b6+
y8+
0
50
150
250
200
400
600
800
1000
1200
